# To Estimate Performance of Artificial Neural Network Model Based on Terahertz Spectrum: Gelatin Identification as an Example

**DOI:** 10.3389/fnut.2022.925717

**Published:** 2022-07-14

**Authors:** Yizhang Li, Lingyu Liu, Zhongmin Wang, Tianying Chang, Ke Li, Wenqing Xu, Yong Wu, Hua Yang, Daoli Jiang

**Affiliations:** ^1^Institute of Automation, Qilu University of Technology (Shandong Academy of Sciences), Key Laboratory of UWB & THz of Shandong Academy of Sciences, Jinan, China; ^2^Shandong Fupai Ejiao, Co., Ltd., Jinan, China

**Keywords:** terahertz time domain spectroscopy (THz-TDS), artificial neural net (ANN), identification, gelatin, multivariate scattering correction, principal component analysis

## Abstract

It is a necessity to determine significant food or traditional Chinese medicine (TCM) with low cost, which is more likely to achieve high accurate identification by THz-TDS. In this study, feedforward neural networks based on terahertz spectra are employed to predict the animal origin of gelatins, whose adaption to the mission is examined by parallel models built by random sample partition and initialization. It is found that the generalization performance of feedforward ANNs in original data is not satisfactory although prediction on trained samples can be accurate. A multivariate scattering correction is conducted to enhance prediction accuracy, and 20 additional models verify the effectiveness of such dispose. A special partition of total dataset is conducted based on statistics of parallel models, whose influence on ANN performance is investigated with another 20 models. The performance of the models is unsatisfactory because of notable differences in training and test sets according to principal component analysis. By comparing the distribution of the first two principal components before and after multivariate scattering correction, we found that the reciprocal of the minimum number of line segments required for error-free classification in 2-D feature space can be viewed as an index to describe linear separability of data. The rise of proposed linear separability would have a lower requirement for harsh parameter tuning of ANN models and tolerate random initialization. The difference in principal components of samples between a training set and a data set determines whether partition is acceptable or whether a model would have generality. A rapid way to estimate the performance of an ANN before sufficient tuning on a classification mission is to compare differences between groups and differences within groups. Given that a representative peak missing curve is discussed in this article, an analysis based on gelatin THz spectra may be helpful for studies on some other feature-less species.

## Introduction

Terahertz techniques are predicted to become promising and influential in the twenty first century. Such view is agreed upon by many researchers (1–3), since a THz wave has unique qualities such as low photon energy, broad band, and high penetrativity for non-polar materials. Additionally, a THz wave bridges microelectronics and optics; the light-matter interaction is also fascinating (4, 5). Terahertz time domain spectroscopy (THz-TDS) is a mature technique for material detection and identification. Potential targets for evaluation are plentiful, including but not limited to explosives, illegal drugs, pharmaceutical, polymers, ceramics, biological tissues, etc. (6–8). Nowadays, an increasing number of investigators focus on the fingerprint THz spectrum of food, and breakthroughs are gradually made with the help of chemometrics and pattern recognition. Generally, identification of a synthetic drug is more available than that of food and traditional Chinese medicine (TCM), because the spectra of purities are in good consistency and tend to show characteristic peaks (non-polar molecules) for identification. For example, one or more peaks are observed in studies on flavonoid compounds by THz-TDS (9), whereas some mineral TCM varieties show similar feature-less THz curves (10). Popular models for THz curve identification include support vector machines (SVMs) (11), artificial neural networks (ANNs) (12–14), partial least squares (PLS) (15–17), random forests (RFs) (18, 19), etc. These models are crucial in utilizing identifiable features that distribute in multiple frequencies and are disturbed by noise from samples and instruments. Tuning is very important for some models like SVMs and ANNs because of their superb capacity in non-linear maps and resultant overfitting.

Slight differences in THz curves resulting from unknown constituent change or difference in mixture have great impacts on difficulties in modeling; thus, black box models are preferred in some studies. An artificial neural network is a representative black box model, which has been sufficiently developed and studied under various backgrounds because of its amazing capacity to make an approximate description of non-linear functions. Among all studies on THz probe of materials, the back-propagation (BP) algorithm is extensively used to adjust the parameters of a network. The name “BP” comes from the fact that the error is propagated backward from the end of the model layer by layer. In every echo, the weight and bias for every cell are updated once. Once the total loss function is smaller enough, the training is completed. The tendency of deploying an ANN model in complex missions is to increase the depth and cell number of models, which would inevitably increase the difficulty for training and the need of numerous samples to avoid overfitting. Considering the frequency resolution, bandwidth, and sample preparation period of THz characterization, a minor ANN model is preferred on a small dataset. Estimating the performance of a model by few sample partitions facilitates target selection and model selection, and avoid excessive parameter tuning. An increasing number of researchers focus on diverse food and medicine, thus, it is also essential to determine identifiable species at low modeling cost as tuning offers limited help for performance lifting of certain model.

Gelatin is a non-toxic natural biomacromolecule comprised of bioactive polypeptides derived from collagen in animal skin, bones, or connective tissues (20), and it is traditionally viewed as restorative or a nutrition supplement in ancient China. Infusion and boiling are the crucial steps for manufacturing gelatin restoratives, which involve complex physical and chemical changes. The particularity in TCM management is material exclusiveness in certain varieties. At present, donkey hide is the specified raw material for *e’jiao* according to Chinese Pharmacopeia, which leads to high market price and fake products made of inexpensive bovine or porcine skin. Modern instruments are highly needed to regulate market conduct by distinguishing *e’jiao* from its major counterfeits. In previous studies, high performance liquid chromatography (HPLC), mass spectroscopy (MS), and real-time quantitative polymerase chain reaction (PCR) have been employed for animal skin identification (19, 21–25). In the abovementioned studies, constituents with different molecules are first separated in space and then detected. For example, chromatography can be further classified according to type of mobile phase or separation mechanism. As a comparison, the THz method, in general, does not require separation or related chemical treatment (i.e., pre-column derivatization in chromatography). THz-TDS is promising to identify gelatins with lower cost and shorter period, while the potential of ANN models in this task is seldom analyzed. An associated question generates that if it is possible to screen recognizable medicines rapidly with ANN models so as to enlarge the scope of multidisciplinary studies. The trial-and-error price is not always welcomed by producer partners of THz researchers who desire to know if the new method is truly good enough for their own production varieties. Therefore, it is also needed to estimate the performance of models in a certain kind with lower cost including reducing the amount of materials needed at experiment level and acceptable period for model optimization.

In this study, we focus on the identification of gelatins using a feedforward artificial neural network (feedforward ANN) and prove that multivariate scattering correction is a simple but effective way to enhance the average performance of feedforward ANNs aimed at gelatin classification. Instead of building a complex model with high accuracy in previous studies (26–29), our study is concerned about the average performance of parallel models with same structure but different dataset partition. Special attention is fixed to describe the impact of input on training models by principal component analysis. It is found that gelatins of different animal origins can be identified with the help of the feedforward ANN and that the key to enhance average performance is to reduce inherent differences within groups. In turn, assessing differences within groups with respect to difference between groups makes it possible to estimate the prospect and adaption of an ANN model on an identification mission.

## Materials and Methods

Gelatin restoratives made of donkey hide, cow hide, or pig skin are newly provided by Shandong Fupai Ejiao Co., Ltd., are packaged with PVC aluminum foil before being used and stored in a medicine cabinet at 4°C and out of direct sunlight. Bulk samples are pulverized and sieved, ending up with powders over 100 mesh. All the powders are compacted to form tablets with diameter of 13 mm (constrained by a mold), mass of 0.2 g (controlled by an electronic balance), and thickness of around 1 mm. Acquisition of spectra is carried out at ambient temperature with moisture below 2%. The number of gelatins is 270 for all and 90 for each type.

**FIGURE 1:**
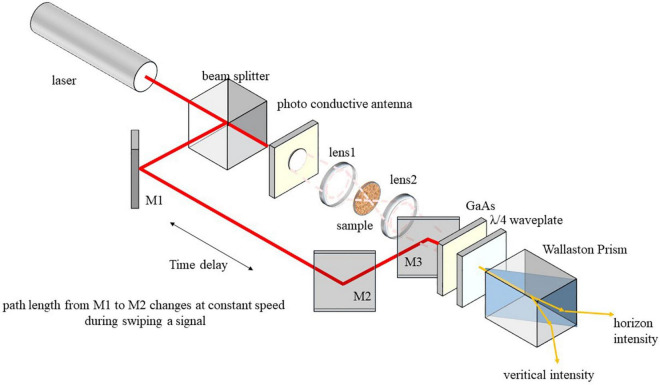
Schematic of terahertz time-domain spectroscopy. It shows the basic optical configuration of a typical terahertz time-domain spectroscopy. Time delay is needed to scan THz pulse with femtosecond laser pulse. Photo conductive antenna is the key component to emit THz wave that is pumped by a femtosecond laser. GaAs is the key component to detect THz wave owing to Pockels effect. Waveplate and Wallaston prism are used to isolate beams with different polarization directions, which are further detected using circuit.


The terahertz time domain spectroscopy device used in this study is a fiber-coupled one from Zomega Terahertz Corporation. The instrument uses a fiber-coupled 1.5-μm pulsed seed laser split into separate amplifiers for a pump and probe beams that are used for THz wave generation and detection. A large-aperture photoconductive antenna is used to generate THz waves, and an electro-optic (EO) crystal is used to detect THz waves (electro-optic sampling). This system measures far-infrared spectroscopy between 0.1 and 3 THz. Peak dynamic range is greater than 1,000 (∼60 dB) for 500 waveform averages (1-s acquisition) and greater than 50 (∼34 dB) at full speed. The schematic of THz TDS is shown in Figure 1. THz TDS provides a rich source of information on a target such as spectroscopic signatures, structural features, thickness, and interfaces of multiple layers. In this study, extinction coefficients derived from the fast Fourier transform of original signals in time domain are the special concern. The relationship between extinction coefficient and frequency spectrum is extensively studied, and details can be found elsewhere (30). A concise formula to calculate the parameter that we used in this study is shown in Eq. 1, where *c* denotes speed of terahertz ray in vacuum, *d* denotes thickness of sample, ρ denotes ratio of amplitude of reference and sample signal, and *n* denotes real part of complex refractive index.

(1) $$\kappa =\frac{c}{\omega \,d}\,l\,n\,\left(\frac{4\,n\,\left(\omega \right)}{\rho \,\left(\omega \right)\,{\left(1+n\,\left(\omega \right)\right)}^{2}}\right)$$

Prior to building artificial neural networks, multivariate scattering correction (MSC) is implemented to reduce errors from scattering. In the algorithm of MSC, each *m* curve is presumed the outcome of a linear function of real spectrum (without error) and the average of acquired data is equal to its expectation. Thus, regression is conducted to adjust the original data, reducing errors that result from gain coefficient (a_*i*_) and bias (b_*i*_). The calculation details can be found in Eqs 2, 3, where *a*_*i*_ and *b*_*i*_ are obtained by least square regression of κ_*i*_ with respect to κ_*real*_.


(2) $$\kappa_{r\,e\,a\,l}=\frac{1}{m}\,\sum_{i=1}^{m}\kappa_{i}$$

(3) $$\kappa_{i\,\left(M\,S\,C\right)}=\frac{\kappa_{i}-b_{i}}{a_{i}}$$

**FIGURE 2:**
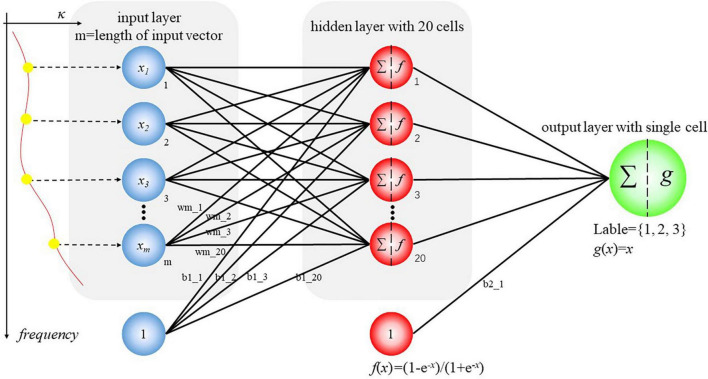
Schematic of a feedforward neural network. A series of universal feedforward neural networks are built, trained, and employed to predict type of gelatin. The values at multiple frequency are viewed as input according to this figure. Besides, this figure also shows the definition of relevant parameters.


Feedforward networks with three levels are employed to identify gelatin groups, where any cell at the hidden level is connected to every cell at the input level and the output level. Besides, there is no direct connection between cells at the input and output levels. A schematic of the feedforward ANN model is shown in Figure 2. In this study, an extinction coefficient between 0.5 and 0.9 THz that contains 91 components is used as an input vector. Initialization of weight and bias is achieved randomly. The models are trained to reduce mean square error (MSE) between output and target by adjusting weight and bias for each node in echoes. The specific training method applied is gradient descent with momentum to avoid local minima. The active function for the hidden layer and the output layer is shown in Eqs 4, 5, respectively. Tuning terminates when maximum gradient is reached (1 × 10^–5^) or MSE is small enough or maximum iteration is reached. The target for training is 1, 2, or 3 according to the animal skin employed in manufacturing. In the beginning, random samples (66% of total) are employed for modeling whose probability to be predicted right in 40 parallel models is expected to be inconsistent. Samples with stable prediction outcome are then marked as curves in part A, and those with unstable prediction outcome were marked as curves in part B. The curves in part A are employed to build additional 20 models for comparison, and their structures are maintained while the parameter is also randomly initialized. Principal component analysis (PCA) is further conducted to compare curves in a feature space with reduced dimensions. In principal component analysis, the value of original data in every dimension is presumed linearly correlated and reconstruction of data in a lower dimension is possible. It is expected that zero-mean input is projected into a new feature whose dimensions are independent, and deviation of original data is reserved as much as possible in new dimensions. The entire approach includes zero-mean normalization, calculation of a covariance matrix and its eigenvectors, and projecting data by matrix multiplication. Suppose the input matrix is *p* in frequency sampling and *m* in sample number, the covariance matrix would be in *p* × *p* size, and the matrix P, made up of eigenvectors, would be in *p* × *q* size, where *q* denotes the number of principal components kept for further analysis. Thus, the output matrix Y is obtained by Y*m* × *q* = X*m* × *p*⋅P*p* × q. Additionally, the variance in a few new dimensions covers a majority of total, and information loss is observed and inevitable because of reducing dimensions. In this study, the first two principal components are utilized for further analysis:

(4) $$y=\frac{2}{1+e^{-2\,x}}-1$$

(5) y=max⁢(x,0)

## Results

**FIGURE 3:**
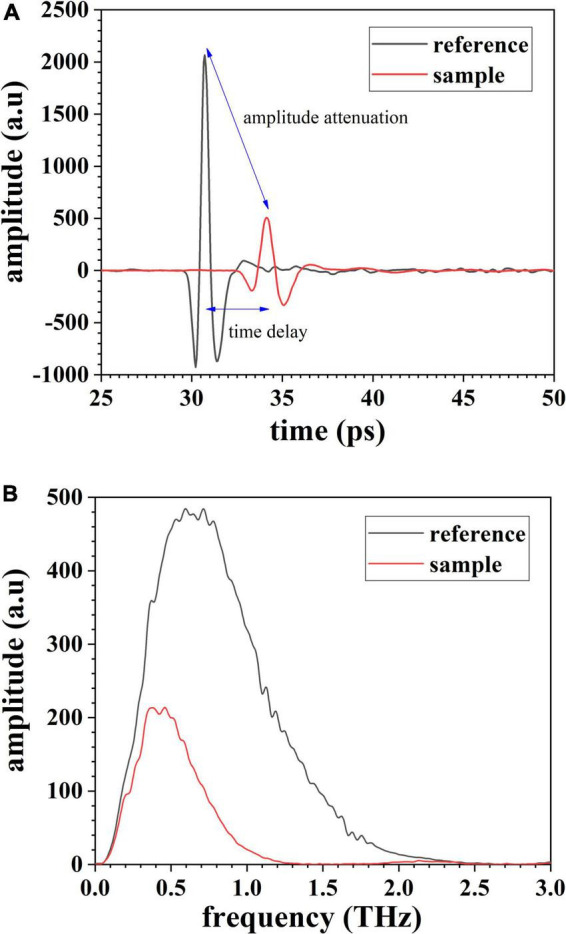
Typical terahertz spectrum of gelatin: **(A)** signal in time domain and **(B)** signal in frequency domain.

A typical spectrum of gelatin in time domain acquired by THz TDS is shown in Figure 3A, and the corresponding frequency spectrum is shown in Figure 3B. As observed in Figure 3A, a pulse with a width of around 1 ps and an amplitude of around 2,000 penetrates the object, resulting in a pulse whose width is broadened and amplitude attenuated. Correspondingly, in Figure 3B, the profile of frequency spectra below 2.5 THz is also changed, and components of the attenuated pulse are in a narrow band of 0–1.3 THz. Such change is mainly attributed to the interaction of photons and molecules of gelatins, in macroscopic view, the absorption. Studies on constituents of gelatins can be found in references, and it is indicated that affirming any constituent of gelatin visually according to THz spectrum is invalid (28, 29). In this case, an algorithm for pattern recognition is needed. As indicated in Figure 3B, peaks from water vapor are not shown, verifying that a test environment filled up with dry air lives up to expectations.

**FIGURE 4:**
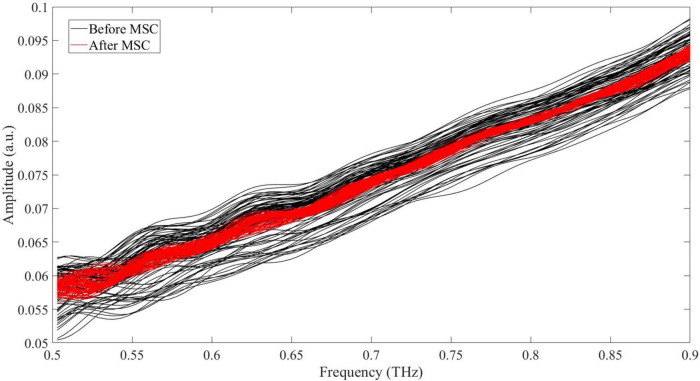
Extinction coefficient of samples in Group 2 before and after MSC.

**FIGURE 5:**
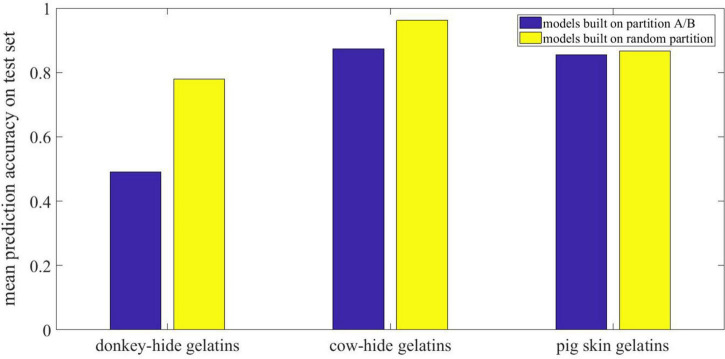
Probability of being predicted right when sample belongs to test set. An ascending sort is given before depicting this figure according to the probability of being predicted right in multiple models for individual sample. The sample number herein is not the coding or order used in tests but the rank according to probability of being predicted right.

The effect of MSC is presented in Figure 4. As is seen, the deviation of samples turns smaller after MSC. The performance of 20 models based on original extinction coefficient and 20 ones based on MSC processed curves is shown in Table 1. As is seen, the models built on MSC-processed optical parameter prevail over the models built on original parameter. Despite good accuracy for training samples, the models built on original extinction coefficient degenerate into unsatisfactory classifiers, especially for donkey hide gelatin and cow hide gelatin. The prediction stability for individual sample is sorted and shown in Figure 5. As is shown, the lowest probability reaches 33.33% (4 out of 12), and 181 samples are predicted right at all times. There are 270 samples in total, among which 66% are randomly picked for training. According to statistics, a sample is trained 36 times at most and 16 times at least, and tested 24 times at most and 4 times at least. It is indicated that almost 66% of samples are not sensitive to dataset partition and parameter initialization.

**TABLE 1:** Accuracy of predictions for models which differ in initialization, sample partition and the samples for training.

Model number	Training set			Test set			Sample partition	Pretreatment to curves
	Donkey-hide gelatins	Cow-hide gelatins	Pig skin gelatins	Donkey-hide gelatins	Cow-hide gelatins	Pig skin gelatins		
1	0.857142857	0.971428571	0.851851852	0.954022989	0.988888889	0.955555556	Random partition	None
2	0.653846154	0.870967742	0.848484848	0.896551724	0.955555556	0.944444444		
3	0.846153846	1	0.815789474	0.954022989	1	0.922222222		
4	0.84375	0.777777778	0.870967742	0.942528736	0.933333333	0.955555556		
5	0.743589744	0.961538462	0.64	0.885057471	0.988888889	0.9		
6	0.821428571	0.965517241	0.909090909	0.942528736	0.988888889	0.966666667		
7	0.807692308	1	0.933333333	0.942528736	1	0.977777778		
8	0.64	1	0.935483871	0.896551724	1	0.977777778		
9	0.766666667	1	0.90625	0.91954023	1	0.966666667		
10	0.76	0.875	0.939393939	0.931034483	0.955555556	0.977777778		
11	0.84	1	0.9	0.954022989	1	0.966666667		
12	0.724137931	1	1	0.908045977	1	1		
13	0.743589744	0.961538462	0.64	0.885057471	0.988888889	0.9		
14	0.909090909	1	0.766666667	0.977011494	1	0.922222222		
15	0.806451613	0.96875	0.888888889	0.931034483	0.988888889	0.966666667		
16	0.677419355	0.914285714	0.958333333	0.885057471	0.966666667	0.988888889		
17	0.814814815	0.96875	0.774193548	0.942528736	0.988888889	0.922222222		
18	0.791666667	1	0.882352941	0.942528736	1	0.955555556		
19	0.8	1	0.928571429	0.931034483	1	0.977777778		
20	0.727272727	1	0.958333333	0.896551724	1	0.988888889		
21	1	1	1	1	1	1	Random partition	Multivariate scattering correction
22	1	1	1	1	1	1		
23	1	1	1	1	1	1		
24	1	1	1	1	1	1		
25	1	1	1	1	1	1		
26	1	1	1	1	1	1		
27	1	1	1	1	1	1		
28	1	1	1	1	1	1		
29	1	1	1	1	1	1		
30	1	1	1	1	1	1		
31	1	1	1	1	1	1		
32	1	1	1	1	1	1		
33	1	1	1	1	1	1		
34	1	1	1	1	1	1		
35	1	1	1	1	1	1		
36	1	1	1	1	1	1		
37	1	1	1	1	1	1		
38	1	1	1	1	1	1		
39	1	1	1	1	1	1		
40	1	1	1	1	1	1		
41	0.512195122	0.923076923	0.861111111	0.770114943	0.988888889	0.944444444	Part A for training; Part B for training	None
42	0.463414634	0.769230769	0.888888889	0.747126437	0.966666667	0.955555556		
43	0.487804878	0.692307692	0.833333333	0.75862069	0.955555556	0.933333333		
44	0.512195122	0.923076923	0.805555556	0.770114943	0.988888889	0.922222222		
45	0.487804878	0.923076923	0.833333333	0.75862069	0.988888889	0.933333333		
46	0.365853659	0.692307692	0.888888889	0.701149425	0.955555556	0.955555556		
47	0.487804878	0.923076923	0.888888889	0.75862069	0.988888889	0.955555556		
48	0.536585366	0.846153846	0.75	0.781609195	0.977777778	0.9		
49	0.512195122	1	0.805555556	0.770114943	1	0.922222222		
50	0.512195122	0.846153846	0.805555556	0.770114943	0.977777778	0.922222222		
51	0.512195122	0.923076923	0.861111111	0.770114943	0.988888889	0.944444444		
52	0.512195122	0.923076923	0.888888889	0.770114943	0.988888889	0.955555556		
53	0.43902439	0.846153846	0.861111111	0.735632184	0.977777778	0.944444444		
54	0.512195122	0.923076923	0.833333333	0.770114943	0.988888889	0.933333333		
55	0.56097561	1	0.833333333	0.793103448	1	0.933333333		
56	0.512195122	0.846153846	0.861111111	0.770114943	0.977777778	0.944444444		
57	0.463414634	0.923076923	0.916666667	0.747126437	0.988888889	0.966666667		
58	0.463414634	0.846153846	0.916666667	0.747126437	0.977777778	0.966666667		
59	0.390243902	0.769230769	0.916666667	0.712643678	0.966666667	0.966666667		
60	0.56097561	0.923076923	0.833333333	0.793103448	0.988888889	0.933333333		

**FIGURE 6:**
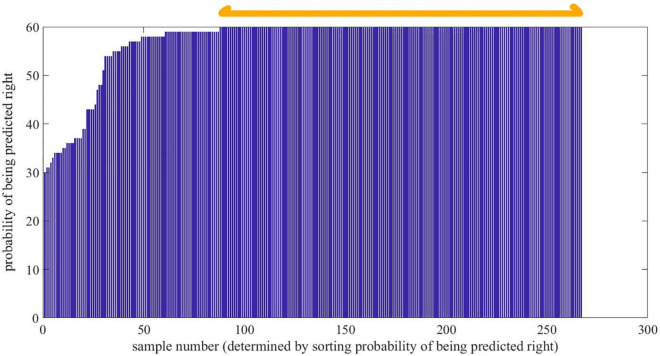
Mean prediction accuracy on test set for special partition and random partition.

Indicated by orange two-way arrow in Figure 5, the original curves in part A are employed to build additional ANN models with the same structure as the ones built already. It is found that the performance in training set reaches 100% whereas the performance in test set is much worse than that of models built on random selected samples without MSC. The performance of each model in three groups is also shown in Table 1. The specific and mean precision for the three groups is shown in Figure 6. As is shown, the performance in training set is still good for all the groups, whereas the performance in test set collapsed because of special sample partition. Figure 6 records the average prediction accuracy under different dataset partitions. Obviously, the models built on general curves prevail over those built on special curves judging from mean prediction accuracy. For donkey hide gelatin, the mean prediction accuracy of models built on general curves reaches 77.9% while such value drops to 49% if certain curves are employed for modeling. Additionally, such drop with respect to cow hide gelatin and pig skin gelatin is not as evident as that for donkey hide gelatin.

**FIGURE 7:**
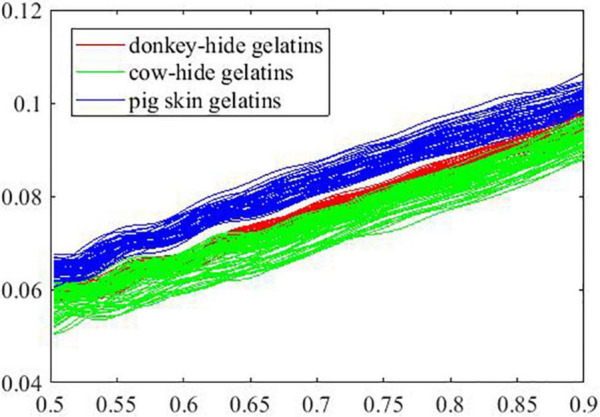
Extinction coefficient of samples in Part A.

**FIGURE 8:**
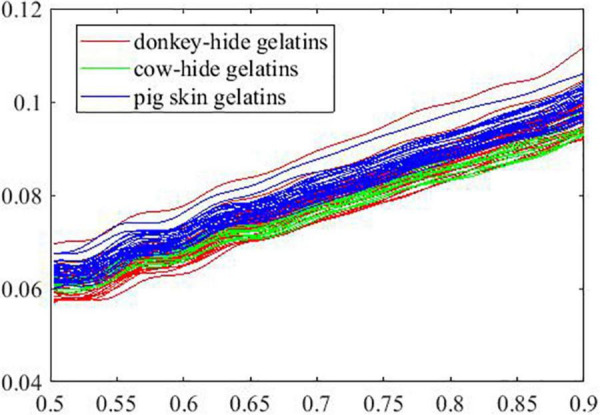
Extinction coefficient of samples in Part B.

The curves in parts A and B are shown in Figures 7, 8 by group, respectively. As is presented, the overlap between curves for samples in different groups is smaller in part A than in part B. In other words, the samples of the three groups in part A are not similar to the samples of the three groups in part B. In Figure 7, curves in blue (pig skin gelatin) are identifiable from curves in red (donkey hide gelatin) and curves in green (cow hide gelatin). On the contrary, a similar phenomenon is not observed in Figure 8.

**FIGURE 9:**
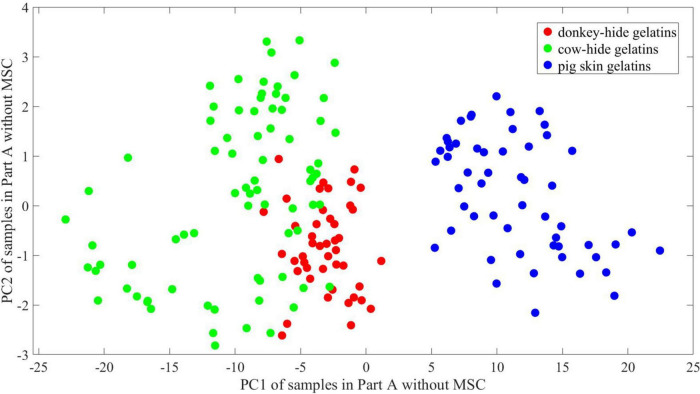
2-D PCA of samples in Part A without MSC.

**FIGURE 10:**
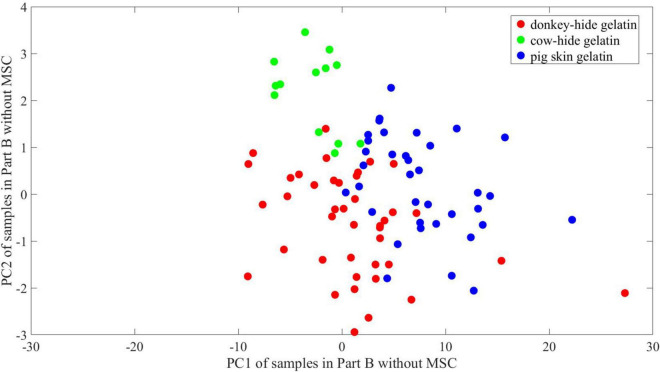
2-D PCA of samples in Part B without MSC.

The explained variance of PC1 and PC2 reaches 95.7 and 2.22%, respectively. The first two principal components of the samples in part A are plotted in Figure 9 where scores of the first principal component (PC1) range from ∼–24 to ∼23, and scores of the second principal component (PC2) range from ∼–3 to 3.3. The border of samples (indicated by convex hull of points in the same group, not depicted) is easy to find with intuition, which suggests good separation between gelatins made of donkey hide and pig skin and gelatins made of cow hide and pig skin. However, the border of donkey hide samples intersects with that of cow-hide samples. Some points lie in the overlapping area, making it impossible to divide them with a line. A similar phenomenon is found in Figure 10 according to points for gelatins made of donkey hide and pig hide. Besides, the area where samples concentrate changes by comparing Figures 9, 10, and its size is also influenced by the number of samples in two parts. The region where points for donkey hide gelatin distribute deviates notably from the one shown in Figure 9 to another one shown in Figure 10.

**FIGURE 11:**
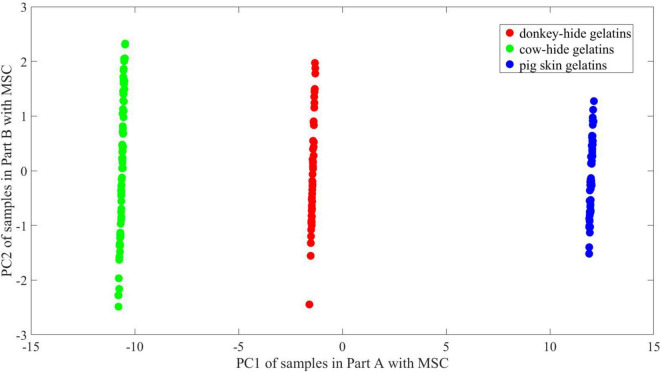
2-D PCA of samples in Part A with MSC.

**FIGURE 12:**
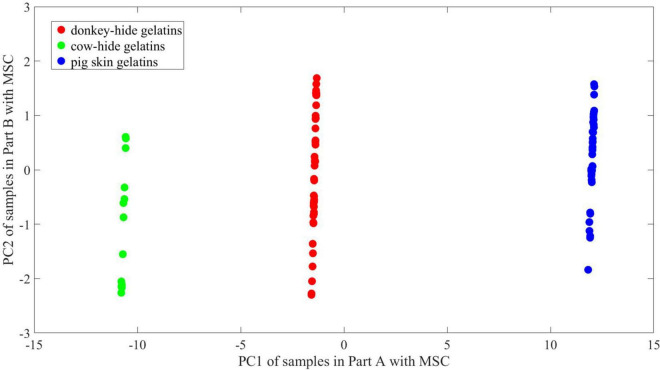
2-D PCA of samples in Part B with MSC.

The explained variance of PC1 and PC2 turns to 96.2 and 1.3% after MSC. As shown in Figures 11, 12, the principal components of gelatins in the three groups align in the direction of PC2. The variation in PC1 for gelatins made of three animals is very limited. Above all, the region where points in different groups concentrate is in nearly the same position according to PC1. Judging from the distribution of PC2, there is also a great similarity between Figures 11, 12.

## Discussion

The THz curves for different gelatins are close to each other because collagen proteins are highly conserved for different species, and gelatins made of different animal skins have common amino acids. For example, glycine, alanine, valine, leucine, isoleucine, and proline are found in gelatins made from donkey hide, while the ratio of these amino acids is different in gelatins made from other animals (31). On the contrary, it is found that gelatins made from different animals have specific peptides to trace their raw materials (15). It is the comprehensive difference in protein instead of specific peptide that causes the difference in THz curve for different gelatins, because a specific peptide is obtained by hydrolysis of protein and further isolated with the help of a chemical agent in LC-MS test. The difference in THz curves of same gelatin is attributed to difference in sample uniformity (including structure and chemical constituents) and measurement error.

Though the structure, training method, active action functions are all maintained for building multiple models, the performance of models on original data varies because the dataset partition is random and parameters of networks are randomly initialized which are further optimized in different paths. As a comparison, the different partition of samples does not have a notable impact on the accuracy of models based on MSC-processed data because the accuracy of prediction maintains 100% for all three gelatins in both sets (see models 21∼ 40). Reasons to employ MSC include ones at physical level and others at mathematic level. THz wave scatters when it interacts with grains in solid materials no matter THz-TDS works in transmittance and reflectance configuration. Such method is also used in studies with FTIR whose structure resembles THz-TDS, as the light path is organized based on Michelson interferometer. Moreover, the obtained spectrum can be expressed by X ≈ aX_0_ in practice, considering the power and delay variation of an input pulse that is generated during tests, where X denotes the obtained spectrum, X_0_ denotes an ideal spectrum, and a denotes a coefficient. Uncertainty in sample thickness also leads to such variation in X according to Eq. 1.

According to the performance of models 41–60, we suppose that some of the original curves are so distinguished from the general ones that the prediction accuracy for the rest drops because of the weak representative curves. Therefore, we record the prediction outcome of all samples in all models and verify our assumption by building additional models with selected samples. According to Table 1, the mean prediction accuracy on test set is generally much lower (shown in Figure 6) if certain curves are employed for building ANNs with the same structure, suggesting that curves in part A are not representative of all samples in a group. Conventionally, a k-fold validation is conducted to solve the problem, and one random partition is not enough to assess the effectiveness of an ANN model. As seen in Figures 7, 8, the overlap between curves for different gelatins is smaller in part A than in part B. Intuitively, samples made from different animals in part A are more identifiable than those in part B. Correspondingly, an attempt to train an ANN model with good generality fails if samples in A are used for training and samples in B are used for testing.

It is hard to describe the similarity in high dimension visually; thus, principal component analysis is employed to illustrate the difference between two parts and the contribution of MSC. We conduct a PCA to show the difference between and within groups in a 2-D space. The change of point distribution from Figures 9–12 releases the reason why MSC is crucial for maintaining high accuracy of models whose training and test sets are randomly partitioned. It is believed that ANNs are equivalent to non-linear function inherently by means of adding non-linear actvie functions and multiple iterations attempting to reduce MSE. As a result, the decision boundary can be an arc at any curvature in the feature space, which matches the dispersive distribution of samples in any space. On the other hand, the parameters of networks are sensitive to irregular distribution of samples. In Figure 11, the 2-D distribution of points for three kinds of gelatins is regular considering PC1 values, and the difference in PC1 between Figures 10, 11 is ignorable. Thus, prediction accuracy can be very high if linear boundary is required in 2-D space. For example, a maximal margin is expected to find final boundary in a vector support machine model (linear SVM), and the mechanism conforms to intuition of mankind. In the 2-D space presented in Figures 9, 10, a linear SVM may have difficulties identifying samples in the overlapping region, and a radial basis function (RBF) core is preferred to achieve higher accuracy. However, a linear SVM adapts to the distribution presented in Figures 11, 12, which may reduce calculation complex. Unlike what occurs in an SVM model, the difficulty in interpreting an ANN model is describing its abstract decision boundary, which is supposed to make sense intuitively in a low-dimension space. Herein, the comparison of Figures 11, 12 suggests that a curve consisting of fewer line segments can be obtained to classify any two gelatins after MSC. In this case, a less-tuned model adapts to the basic need of spectrum identification. Therefore, the relatively simple structure of an ANN can be promising in specific missions regarding THz-TDS, because the obtained THz spectrum lies in a limited neighborhood of an ideal spectrum owing to good consistency during sample preparation and the high signal noise ratio of THz-TDS. The analysis of an ANN with parallel models, PCA, and MSC roots in basic thought of linear discrimination analysis (LDA). By maximizing *J* in Eq. 6, which is named generalized Rayleigh quotient, one expects to find an optimal projection. The definition of *S*_*b*_ and *S*_*w*_ is seen in Eqs 7, 8, where x is the input vector, X_0_ and X_1_ represent two groups, and μ_0_ and μ_1_denote the mean of x in X_0_ and X_1_ respectively. A mathematical expression with respect to S_*b*_/S_*w*_ is proposed to describe the identifiability of data in a 2-D space using a line. In 2-D space, certain distribution of points is not welcome, as convex hulls of points in different groups overlap. In this case, further projection of data is essential and preferred in some methods including SVM, which is achieved using core function. The decision boundary is hard to interpret for ANNs in 2-D space. In order to describe linear separability of data in 2-D space and possible decision boundary, we assume that at least *s* straight lines or line segments can form a boundary set that separates samples in different groups with no error. The boundary can be a combination of polygons and polygonal lines in shape and whose total number is no bigger than s. The reciprocal of *s* is viewed as the index to reflect linear separability of data. According to Figures 11, 12, one straight line is enough to achieve good accuracy, whose slope and offset lie within an interval. Correspondingly, the proposed linear separability in 2-D principal component space equals 1 for every two kinds of gelatin. In Figures 9, 10, more line segments are required to bypass points in the overlapping area of the three convex hulls. In this case, the linear separability is lower than 1 according to our definition. The linear separability of data determines if the ANN model requires harsh training from certain initial points according to specific training function. In another view, the linear separability of data also determines if an ANN model with more complex structure (more levels) is needed. Considering that maximum linear separability can be obtained after MSC, we obtain a good accuracy in models 21–40.


(6) $$J=\frac{w^{T}\,S_{b}\,w}{w^{T}\,S_{w}\,w}$$

(7) $$S_{w}=\sum_{x\in X_{0}}\left(x-\mu_{0}\right)\,{\left(x-\mu_{0}\right)}^{\text{T}}+\sum_{x\in X_{1}}\left(x-\mu_{1}\right)\,{\left(x-\mu_{1}\right)}^{\text{T}}$$

(8) $$S_{b}=\left(\mu_{0}-\mu_{1}\right)\,{\left(\mu_{0}-\mu_{1}\right)}^{\text{T}}$$

The fluctuation in prediction accuracy with respect to sample partition and parameter initialization in tests without MSC proves that it is hard to train an ANN model with good generalization performance unless the difference within the same group is effectively weakened compared to the difference between groups. In other words, a study shall be focused on model selection rather than parameter tuning on multiple partitions only if the random partition of a training set and a test set is representative enough. Therefore, we must consider chemical uniformity and material dispersibility during sample preparation. In this study, the stickiness of gelatin makes it difficult to obtain fine powder by grinding and screening but also makes it possible to omit an agglomerant (usually high-density polyethelene, HDPE), which is needed in some cases. Thus, we do not concern about extra scattering effect from different size and different distribution of two grains in a tablet. The THz curve in gelatin studies is representative of several foods that do not show peaks because of complex chemical constituents (16,32). The reason for optimizing an ANN with complex structure in similar studies is to balance fit and overfit. However, the upper limit of outcome is inherently determined by the individual difference of species or the consistency of process. It is indicated in this study that by comparing the difference between groups and difference within groups, one can estimate the performance of an ANN by random initialization and sample partition. As a result, one can double attempts for characterizing more species using an ANN with same structure.

As a booming technique to characterize object, an increasing number of investigators would focus on multidisciplinary studies using THz TDS as a booming technique and develop new models for better identification of diverse food and medicines. It seems that the combination of pattern recognition and THz curve underlies the universal application of THz-TDS in the food and medicine industry. The distribution and shape of a THz curve, rather than molecular weight, are the key factors to select appropriate models. However, challenges apart from algorithm also make it tough to conduct THz-TDS in field. First, the THz method is not a real-time technique presently for monitoring production, because a physical process is conducted to prepare samples in almost all studies. The method for preparing samples described in this manuscript adapts to only a part of TCM species or foods. Another notable limitation of the THz method is its lower analytical precision compared to that of chromatography, since separation of materials is not conducted before detection on tests in THz-TDS. Thus, an unknown slight change in certain chemical components may cause variations in spectrum. Trace detection for pure substances based on THz-TDS can be fulfilled with the help of a metamaterial, which is not used herein because gelatins contain various chemical components. Besides, complete separation of gelatin and metamaterials is the prerequisite for conducting tests with acceptable cost but has not been well-studied.

## Conclusion

Gelatins made from three different animals are measured by THz-TDS in this study, and extinction coefficients between 0.5 and 0.9 THz are employed for training feedforward neural networks. The adaption of feedforward neural networks is examined by random sampling for training and parameter initialization with random values. Sixty parallel models are built to study the estimation of ANN performance where 20 are built on original curves, 20 are built on MSC processed curves, and 20 are built on original curves after special partition. It is found that the generalization of feedforward ANNs is improved by MSC. It is found that the reciprocal of minimum number of line segments required for error-free classification in 2-D feature space can be viewed as an index to describe the linear separability of data. The rising of proposed linear separability would lower the requirement for harsh parameter tuning of ANN models and tolerate random initialization. The difference in principal components of samples between training sets and data sets determines whether partition is acceptable or whether a model would have a good generality. A rapid way to estimate the performance of an ANN before sufficient tuning on a classification mission is to compare the difference between groups and the difference within groups, which may facilitate the popularization of THz-TDS in probing additional varieties.

## Data Availability Statement

The datasets generated during and/or analyzed during the current study are available from the corresponding author on reasonable request.

## Author Contributions

YL: conceptualization, methodology, software, and writing (original draft). LL: conceptualization, methodology, formal analysis, and writing (review and editing). ZW: methodology, visualization, and project administration. TC: methodology. KL: validation, formal analysis, resources, and funding acquisition. WX: validation, resources, and funding acquisition. YW: investigation and resources. HY and DJ: resources. All authors contributed to the article and approved the submitted version.

## Conflict of Interest

YW, HY, and DJ were employed by Shandong Fupai Ejiao, Co., Ltd. The remaining authors declare that the research was conducted in the absence of any commercial or financial relationships that could be construed as a potential conflict of interest.

## Publisher’s Note

All claims expressed in this article are solely those of the authors and do not necessarily represent those of their affiliated organizations, or those of the publisher, the editors and the reviewers. Any product that may be evaluated in this article, or claim that may be made by its manufacturer, is not guaranteed or endorsed by the publisher.
